# Greater Gender Diversity Observed at Orthopaedic Conferences in the Caribbean Than in the United States or England

**DOI:** 10.7759/cureus.28224

**Published:** 2022-08-21

**Authors:** Marlon M Mencia, Shanta Bidaisee, Camille Quan Soon, Shamir O Cawich

**Affiliations:** 1 Surgery, The University of the West Indies, St. Augustine, TTO; 2 Surgery, The Port of Spain General Hospital, Port of Spain, TTO

**Keywords:** the university of the west indies, the caribbean association of orthopaedic surgeons, residents, orthopaedic surgery, gender diversity

## Abstract

Introduction

Women are underrepresented in orthopaedics. Recent studies have shown that women comprise only a very small proportion of all practising orthopaedic surgeons in the United States. One theory that seeks to explain this disparity is the lack of female mentors in orthopaedic surgery. Women are particularly influenced by same-sex mentors, and the paucity of mentors sets up a negative feedback loop that further reduces applications to residency programs. Presentation of scholarly work at conferences increases the visibility of women and represents important opportunities to encourage young female doctors to the speciality. The annual meeting of The Caribbean Association of Orthopaedic Surgeons (TCOS) is a forum that allows regional exposure to young doctors. In the present work, we aim to analyse the gender diversity among presenters at the annual TCOS meetings.

Methods

A retrospective analysis of the final programs of TCOS meetings over five years was conducted to determine the sex of the presenter, their roles, and topics. The first author listed on the program was taken as the presenter unless it was otherwise stated. Gender was determined using conventional naming taxonomy. Presenters were classified as podium presenters or moderators and presentations as clinical or non-clinical. We subdivided clinical presentations into seven subspecialty areas. A Chi-squared test was used to calculate differences between groups, with a p-value of < 0.05 representing significance. Statistical tests were performed using Analyse-it for Microsoft Excel 5.40 (Analyse-it Software Ltd).

Results

There was a total of 195 podium presentations over the study period. During this time, there was a steady increase in the number of presentations by women, with a mean value of 19.5%. Compared with men, women presented fewer clinical papers (52.6%vs 73.9%, p=0.037), presented on more general topics (63.2% vs 31.2%, p=0.007) and were less likely to moderate a session (2.7% vs 97.3%, p=0.014). Presentations by female residents were marginally higher than the mean rate for women overall and were representative of the gender distribution in the resident pool.

Conclusions

There are significantly fewer presentations by women than men at the annual TCOS meetings. Encouragingly, however, there is a positive trend towards greater female presentations over the study period. Our results show that despite being underrepresented, more women are presenting at orthopaedic conferences in the Caribbean than in the United States or England.

## Introduction

A competent gender-diverse workforce is an essential part of any modern healthcare system. As leaders, women have expanded marketing, enhanced institutional creativity and improved decision-making [1]. However, despite the many benefits of gender inclusivity, women remain under-represented in healthcare and more directly in academic medicine. Recent data show a significant gender gap in surgical residency and, in particular, orthopaedic surgery [2]. Currently, in the United States (US), 25% of residency programs have either one or no female residents.

Furthermore, women form only 13% of US orthopaedic residents and just 8% of practising orthopaedic surgeons [3,4]. Limited exposure to orthopaedic surgery, in addition to few female mentors, has been suggested to contribute to the current gender gap [5-9]. Same-sex role models are important to women, and greater exposure to female orthopaedic surgeons, including residents, is likely to encourage other women to enter orthopaedics [5,8-11].

Podium presentation at academic conferences is one outlet that permits young female academics to highlight their scholarly work and build a national reputation. Both are important for academic promotion and tenure. Current trends indicate that more women present at national orthopaedic meetings but at significantly lower rates than men [12,13]. Studies of the British Orthopaedic Association (BOA) meeting and the American Academy of Orthopaedic Surgeons (AAOS) meeting reveal that women presented only 10.2% and 10.6% of the papers, respectively [12,13]. As invited speakers, women fared marginally better. In a cross-sectional study, Gerulli et al. found that women comprised 14% of all invited speakers but were more likely to have nontechnical speaking roles than men [14].

Assessment committees frequently use podium presentations to measure research productivity which influences academic promotion. It follows, therefore, that gender disparity at national meetings may contribute to the small number of women in senior faculty positions. This is particularly concerning since women are more influenced by same-sex role models when deciding to pursue a career in orthopaedics [5,8-10,15]. Without exemplars, female medical students may be reluctant to enter orthopaedic residency, creating a self-perpetuating cycle that further widens the gender gap in orthopaedics.

A literature search revealed a lack of data from the Caribbean focusing on gender diversity in healthcare. Furthermore, there are no studies looking specifically at orthopaedic surgery. Our research seeks to fill a gap in the literature by analysing the gender diversity in the region, using data collected over five years from the annual meeting of The Caribbean Association of Orthopedic Surgeons (TCOS). This article was previously presented as a virtual podium presentation at the 2021 Caribbean Association of Orthopaedic Surgeons Annual Scientific Meeting on November 20, 2021.

## Materials and methods

**Figure 1:**
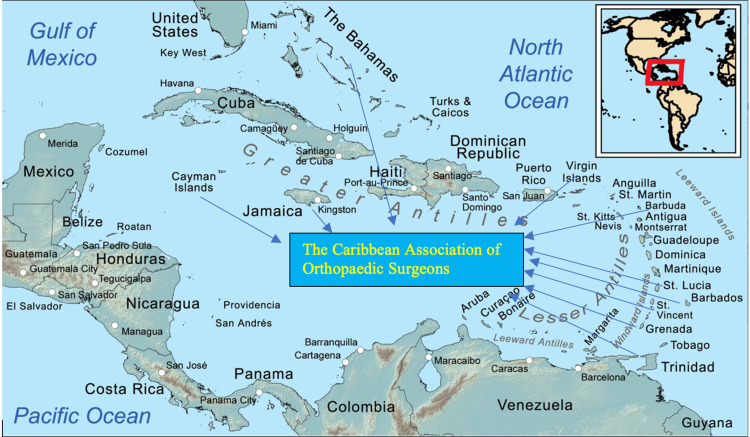
Member islands of the Caribbean Association of Orthopaedic Surgeons. Kmusser (https://commons.wikimedia.org/wiki/File:Caribbean_general_map.png), "Caribbean general map", https://creativecommons.org/licences/by-sa/3.0/legalcode

The Caribbean Association of Orthopaedic Surgeons (TCOS) is a professional association of orthopaedic surgeons across the Anglophone Caribbean (Figure 1).

Its mandate is to promote the science, art and practice of orthopaedic surgery in the region. This is achieved by hosting an annual three-day academic symposium that focuses on education and research. During the symposium, members and invited guests participate in podium presentations, panel discussions and surgical workshops.

Permission was obtained from TCOS to access an electronic register of academic programs from the annual symposia from 2016 to 2020. Due to the COVID-19 pandemic, the 2020 meeting was held virtually, although its format was unchanged. Independent researchers retrospectively evaluated the programs, and the following data were extracted: participant category (moderator or podium presenter), presentation area (clinical vs non-clinical) and presenter gender. The first author listed on the program was assumed to be the presenter unless it was explicitly stated that another author was presenting. Gender was determined using conventional naming taxonomy, but in cases of ambiguity, this was confirmed by the TCOS organising committee. Using this method, the gender of all presenters was established.

Two categories of speaking opportunities were identified based on the level of prestige. Podium presenters were speakers who were selected after a blinded peer review of their submitted abstracts to present at the annual symposia. Moderators were invited to lead a session and stimulate post-presentation discussion and were selected by virtue of their reputation and subject-area expertise.

Presentations were either clinical or non-clinical. Clinical presentations included case reports, case series, and descriptions of surgical techniques and/or complications. These were further subdivided into seven subspecialty areas, including trauma, adult reconstruction, paediatrics, oncology, sports medicine, and spine. Because of the small numbers, anatomical regions, e.g., shoulder, elbow, foot and ankle, were broadly grouped into sports medicine. A general category was added to include clinical topics that did not fit neatly into subspecialty areas, such as infections. Non-clinical topics focused on medical education, basic sciences and medical ethics. Our primary outcome was to determine the percentage of female presentations and secondarily to analyse the types of presentations and the roles of women at the conference.

This study did not require ethical approval since it is considered nonhuman research and met the criteria for Waiver of Review by The School for Graduate Studies and Research of The University of The West Indies. Data were collected in Excel, and descriptive statistics were used to illustrate the results. The percentage of female presenters, the role of the presenter, presentation type and clinical subspecialty type were calculated, and data were compared between men and women. We used a Chi-squared test to evaluate differences between groups, and a p-value of < 0.05 was considered significant. Statistical tests were performed using Analyse-it for Microsoft Excel 5.40 (Analyse-it Software Ltd).

## Results

**Figure 2:**
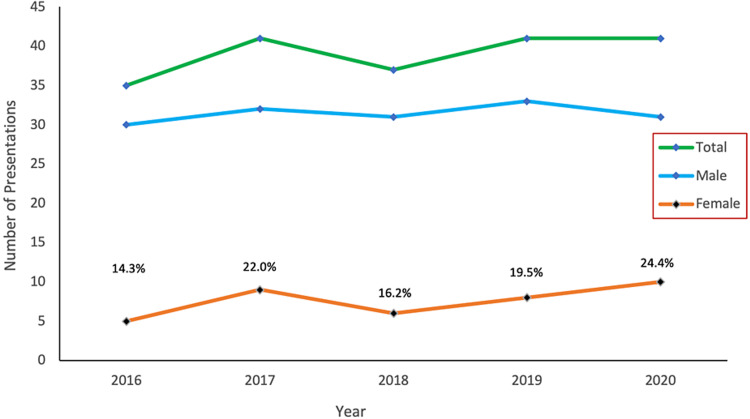
Total number of presentations and presentations by male and female speakers at the Caribbean Association of Orthopaedic Surgeons meetings 2016–2020.

A total of 195 podium presentations were given at the annual TCOS meetings between 2016 and 2020. The number of presentations did not change significantly over the five years (Mean 39, Range 35 - 41, p = 0.9357). While the percentage of female presentations increased over the study period (Mean 19.5%, Range 14.3%-24.4%), men still delivered the majority of presentations (80.5%). The number of presentations per year based on sex is shown in Figure 2.

Clinical vs non-clinical presentations

**Figure 3:**
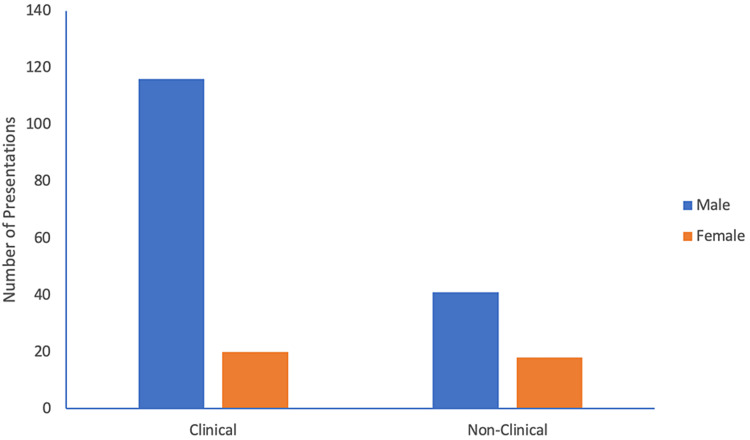
Categories of presentations by both male and female presenters.

The annual meetings of TCOS are primarily clinically oriented with a 70:30 ratio (clinical: non-clinical presentations). Men were more likely to present clinical topics than women, delivering five times as many clinical presentations as women (M:F, 116:20) but only twice as many non-clinical presentations (M:F, 41:18 p=0.037) (Figure 3).

Subspecialty presentations

**Figure 4:**
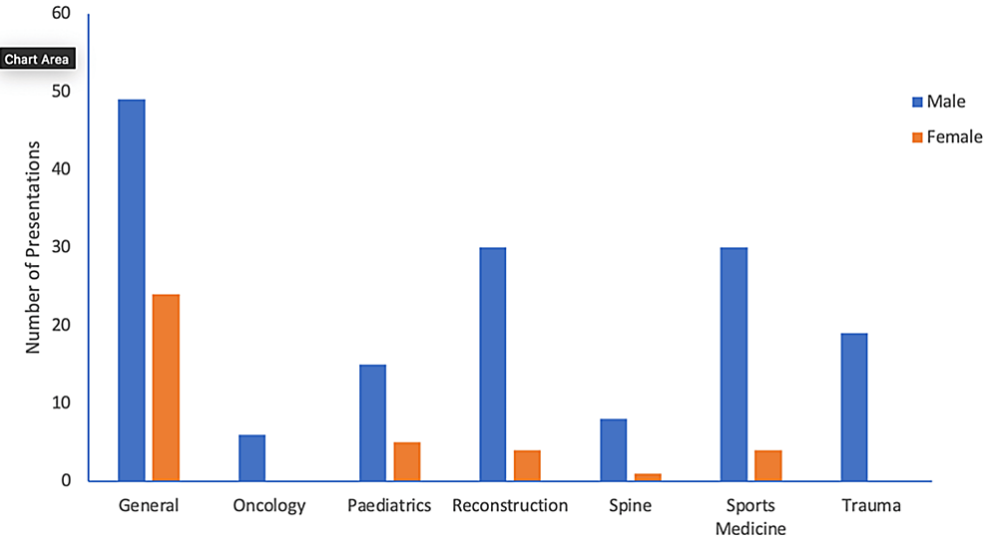
Subspeciality presentations by both male and female presenters

In all subspecialty categories, the number of presentations by men was greater than women, with no female presentations in oncology and trauma and only one in the spine. There was a scarcity of female representation in the popular subspecialty areas, with only four presentations in both adult reconstruction and sports medicine (11.7%, 4/30). The majority of presentations by women were in general category (63.2%, 24/38) (Figure 4).

Role of the presenters

**Figure 5:**
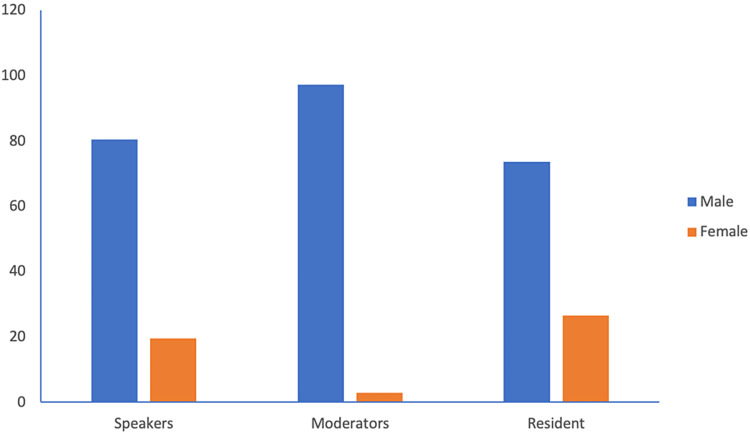
Percentage of men and women in different roles.

Compared with men, women were less likely to preside over a forum, and women moderated only one session (2.7%, 1/36). There was a statistically significant difference for women when podium presenter and moderator roles were compared (Podium presenter v moderator; 38 v 1; p=0.014) (Figure 5).

Resident presentations

**Figure 6:**
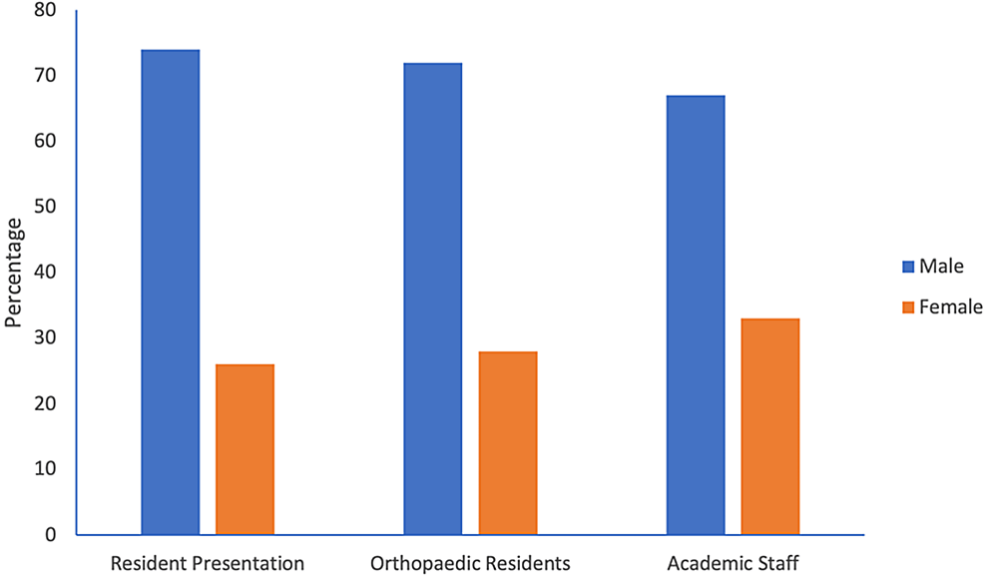
The gender composition of resident presentations compared with the gender composition of the orthopaedic residents and academic staff at the UWI.

There were 34 resident presentations over the five meetings, of which nine (26.5%) were delivered by women. The gender distribution of resident papers closely mirrored that of the resident pool and academic staff (Figure 6).

## Discussion

Academic faculty and residents benefit from presenting their research at annual conferences as the first step towards publication. While several studies have shown that women publish at lower rates than their male colleagues, more recently, it has emerged that women are also less likely than men to present their work at annual conferences [12-14].

Our results show that women delivered 19.5% of the presentations at TCOS, and we were encouraged to find that the proportion of female presenters increased annually from 14.3% to 24.4%. This compares favourably with similar studies from the United States and England. In the United States, between 2008 and 2017, female presenters at the American Academy of Orthopaedic Surgeons (AAOS) increased marginally from 6.6% to 10.6% [12]. More concerning, Krahelski et al. reported that female presentations at the British Orthopaedic Association (BOA) actually fell from 13.3% in 2014 to 11.7% in 2017 [13]. A more in-depth analysis of our data shows that female presentations at TCOS grew at an annual rate of +2.53%, while the rate at the AAOS was only +0.45%, and female presentations at the BOA declined at an annual rate of -0.53%. Although TCOS is a small and new organisation compared with the BOA and the AAOS, the relatively high percentage of female presentations and positive annual growth are very promising.

A closer look at our data revealed some important differences in the types of presentations and the role of the presenter between men and women. We reported that compared with men, women presented fewer clinical papers (52.6%vs 73.9%), more general topics (63.2% vs 31.2%) and were less likely to moderate a session (2.7% vs 97.3%). While these results are disturbing, they are not unique, and similar findings have been reported by several studies [12-14]. Krahelski et al. found that men presented twice as many clinical topics as women (42.4% vs 20.7%) while the most common session for female presenters was education. Furthermore, among the clinical topics, there were no presentations by women in oncology, one in the spine (0.9%) and seven in trauma (4.7%) [13]. Along the same lines, Tougas et al. have shown that women were less likely than men to be included as faculty or moderators at national orthopaedic meetings [12]. The reasons for the such disparity are beyond the scope of our study. Implicit bias, either at the individual or institutional level, has been cited as a contributing factor [15,16]. Denying women the opportunity to contribute at a higher level and confining their involvement to less prestigious roles is a form of implicit bias and prevents their full inclusion [12,14].

To the best of our knowledge, this study is the only one to analyse the gender of resident presenters. Our results show that female residents presented 26.5% of all resident presentations over five years. This finding is significant for two reasons. First, the rate is higher than the female presentation of the general faculty. Second, it also shows that women are presenting at rates which are proportional to the gender ratio of the training program. Both reasons imply that applications by female residents to present at orthopaedic conferences are not being overlooked. Female role models are important to women when evaluating orthopaedic residency programs and lead to greater numbers of applications to the field [9,10,17]. However, this leadership role should not be confined to consultant-level surgeons but also extend to the residents in training. It is postulated that female medical students, who perceive orthopaedic surgery as a male-dominated speciality can more readily identify with a same-sex resident than a consultant. In this way, female residents act as “trailblazers”, encouraging greater recruitment of women into the speciality [5,8,18]. Therefore, if we are to narrow the gender gap in orthopaedics, it is essential that greater support is afforded to female resident presentations at national meetings.

Increasing the number of female orthopaedic surgeons is an important factor in providing a better specialist service. A more gender-diverse workforce allows patients greater choice when choosing a physician. The gender preference of patients for same-sex physicians is firmly accepted in general practice, and a recent study suggests that this is also true for surgical specialities, including orthopaedics [19]. Furthermore, there is evidence to suggest that better surgical outcomes are achieved when patients are treated by physicians of the same sex [20]. Most compellingly, however, is the urgent need to recruit women to bolster the already insufficient orthopaedic workforce that is predicted to be placed under further pressure caused by retirement and the demands of an ageing population.

There are several limitations to our study. While we were careful to ensure that the final conference program reflected the actual presentations at the conference, we could not exclude that there may have been last-minute changes to the topic or presenter which could have affected our data. Accurate categorization of the presentations is important; however, it is possible that some presentations would have qualified for more than one category. To offset this, two authors independently categorised the presentations and resolved discrepancies through consensus to reduce the impact of subjectivity on the results. Finally, because of the small numbers in some categories, we were compelled to pool data to allow for meaningful statistical analysis.

## Conclusions

Orthopaedic surgery is widely regarded as a male-dominated speciality and has one of the lowest percentages of female surgeons among surgical subspecialities. Therefore, we were encouraged by the results of our study, which show higher levels of female presenters at TCOS compared with similar conferences in the United States and England. Although there remains significant work to be done before gender parity can be realised in orthopaedics, the trend observed in this study is reassuring. Women should be encouraged to pursue a career in orthopaedics through greater exposure and representation at national conferences.
